# Adherence to Exclusive Breastfeeding and Associated Factors in Mothers of HIV-Exposed Infants Receiving Care at Kilimanjaro Christian Medical Centre, Tanzania

**DOI:** 10.24248/EAHRJ-D-16-00365

**Published:** 2018-04-01

**Authors:** Murtaza Husain Rasheed, Rune Philemon, Grace Damas Kinabo, Maya Maxym, Aisa Mamuu Shayo, Blandina Theophil Mmbaga

**Affiliations:** a Department of Paediatrics and Child Health, Kilimanjaro Christian Medical University College, Moshi, Tanzania; b Department of Paediatrics and Child Health, Kilimanjaro Christian Medical Centre, Moshi, Tanzania; c Kapi'olani Medical Center for Women and Children, John A. Burns School of Medicine, University of Hawai'i at Manoa, Honolulu, Hawaii, USA; d Kilimanjaro Clinical Research Institute, Moshi, Tanzania

## Abstract

**Background:**

More than 90% of HIV in children occurs through mother-to-child transmission. Breastfeeding is one of several factors associated with transmission of HIV, and, because of this, infant feeding practice is one of the cornerstones in the effort to reduce HIV transmission in children. World Health Organization guidelines from 2012 recommend exclusive breastfeeding to all infants for first 6 months of life. However, many factors contribute to the adherence of mothers to exclusive breastfeeding (EBF) recommendations. The aim of this study was to determine what factors likely influence adherence to exclusive breastfeeding in mothers of HIV-exposed infants receiving care at Kilimanjaro Christian Medical Centre.

**Methods:**

A cross-sectional hospital-based study was conducted from September 2012 to March 2013 at Kilimanjaro Christian Medical Centre in Moshi. All mothers of HIV-exposed infants aged 6 to 12 months receiving care at child-centred family care clinic who consented to participate in the study were included. Data were collected using a pretested structured questionnaire and analysed using SPSS version 16.0 statistical package.

**Results:**

Of the 128 mothers of HIV-exposed infants enrolled in the study, 71 (55.5%) adhered to EBF for 6 months. No statistical significance was seen between adherence status and maternal characteristics in bivariate analysis (*P*>.05). The mean age and standard deviation of initiating other foods by mothers who did not adhere was 3.32 months ± 1.24. Of 57 (44.5%) non-adherent mothers, one-tenth of them practised mixed breastfeeding and the rest stopped breastfeeding completely. Fear of postnatal transmission of HIV through breastfeeding and inadequate breast milk production were the most common reasons for non-adherence to EBF.

**Conclusion:**

Adherence to the recommended duration for EBF by mothers to their HIV-exposed infants is still a challenge. Ongoing intensive feeding counselling and education on EBF may increase the number of mother who can adhere to EBF in our society.

## BACKGROUND

In 2011, about 330,000 children, approximately 1,000 each day, were newly infected with HIV, more than 90% of whom were from sub-Saharan Africa.^1^ Since 2010, significant efforts have been made by prevention of mother-to-child transmission (PMTCT) programmes to reduce the transmission of HIV in children in low- and middle-income countries (LMICs).^2^ A recent study in Moshi, Tanzania, using birth registry data from mothers with known HIV status, showed the percentage of infants who tested HIV positive declined from 17.4% in 2000 to 4.0% in 2014,^3^ and the proportion of mothers who knew their HIV status at the time of delivery increased from 5.4% in 2000 to 98.8% in 2014.^3^

Globally, over 90% of children acquire HIV through mother-to-child transmission (MTCT) as reported in 2010.^4^ Mothers living with HIV, who are not virally supressed, can transmit the virus during pregnancy, labour, delivery, and breastfeeding. Without any intervention, the risk of MTCT is 15% to 45%, while transmission through breastfeeding alone ranges from 5% to 20%. However, with PMTCT interventions the risk of MTCT of HIV reduces to 1% to 15%, while MTCT through breastfeeding reduces risk to less than 5%.^5^ The critical component of an effective PMTCT program is infant-feeding counselling and practice. Therefore, a primary focus for reducing MTCT of HIV is through infant feeding practices; this is especially true in LMICs, especially in the breastfeeding communities.^6^

In 2012, the World Health Organization (WHO) recommended exclusive breastfeeding (EBF) of infants for the first 6 months to all mothers, regardless of the HIV status, and emphasized this recommendation for mothers living with HIV.^7^ In contrast, mixed breastfeeding (MBF) has a fourfold higher risk of postnatal transmission of HIV compared to EBF.^8^ Additionally, replacement feeding is recommended only when it is acceptable, feasible, accessible, sustainable, and safe (AFASS), which is rarely the case in a resource-limited setting.^7^ Despite WHO recommendations, with Tanzanian mothers living with HIV, the mean age for cessation of EBF was 3 months.^9,10^ A majority of mothers living with HIV who initially choose to practice EBF did not adhere to it for the entire first 6 months, usually reverting to MBF until weaning. This increases the risk of HIV transmission to the infant.^11^ For PMTCT programmes, EBF for 6 months before weaning is crucial for reducing risk of HIV infection in infants. Many factors contribute to the adherence of EBF by mothers living with HIV. The 2016 WHO breastfeeding guidelines updates the 2010 guidelines to state that breastfeeding should not to be restricted in settings where health services provide and support lifelong ART and adherence counselling. While emphasizing EBF for 6 months, breastfeeding should be promoted and supported for at least 12 months, and women may continue breastfeeding for up to 24 months or longer, similar to the general population.^12^

The aim of this study was to determine the proportion of mothers living with HIV adhering to EBF for 6 months and the factors that influence adherence to EBF. This information could be used by concerned bodies to improve feeding counselling strategies in order to reduce MTCT of HIV.

## METHODS

### Study Design

This was a cross-sectional hospital-based analytical study carried out at Moshi municipality in Kilimanjaro region at child-centred family care clinic of Kilimanjaro Christian Medical Centre (KCMC) in the northern zone of Tanzania. Moshi municipality has an estimated population of about 200,000 people. The study was conducted for 6 months – from September 2012 to March 2013. KCMC was selected because of its long-standing PMTCT clinic and experience in feeding counselling.

### Study Population

All mothers with HIV-exposed infants aged 6 to 12 months who opted for EBF, were receiving care at the HIV-exposed baby follow-up clinic at KCMC, and gave informed consent to participate in the study were eligible for enrolment. Mothers who opted for alternative feeding (formula feeding or cow's milk) or mixed-feeding options or breastfeeding mothers who did not sign consent for participation were excluded from the study.

### Sample Size and Procedure

The minimum sample size was estimated using a formula by the Survey System (Creative Research Systems, Sebastopol, CA, USA) and Joint WHO and DGIS (1988) expressed as sample size (SS) = Z^2^P(100-P)/∈^2^, where, Z is the value (1.96 for 95% confidence level [CI]), P represents prevalence, and ε is the minimal tolerable error at 95% CI, expressed as a decimal (0.05). A prevalence of 13.3% for EBF was selected based on a study done by Young et al.^10^ The minimum estimated sample size was 178 mothers. Of the 157 mothers living with HIV with infants born between October 2011 and September 2012 and received care at KCMC, only 128 opted for EBF and consented to be recruited for this study.

### Operational Definitions

At the time of this study, the following WHO definitions were used:^13^

- *Exclusive breastfeeding* is defined as giving ONLY breast milk, except for prescribed medicines, vitamins, and mineral supplements for the first 6 months of life.
- *Mixed breastfeeding* is defined as giving breast milk and ALSO other liquids such as water, tea, formula, cow's milk, or foods such as porridge or rice for the first 6 months of life.
- *Replacement feeding* indicates that the infant NEVER receives breast milk but instead is given a breast-milk substitute, such as commercial infant formula or modified animal milk for the first 6 months of life.

### Data Collection Procedure

Data were collected using a structured questionnaire by the principal investigator who also identified the mothers who opted for EBF during the clinic visit. The questionnaire included questions about sociodemographic characteristics, factors that likely influence adherence to EBF, and barriers for non-adherence to EBF, and was pretested for comprehension and accuracy. Respondents were interviewed in Kiswahili language.

### Data Processing and Analysis

Data were cleaned, manually entered, and analysed using Statistical Package for the Social Sciences for Windows version 16.0 (SPSS Inc., Chicago, IL, USA). Univariate and bivariate tables and multiple logistic regressions were used to investigate associations between the outcome and different determinants. Data were summarized into frequency tables, bar charts, pie charts, and percentages of different variables related to the outcome. Factors found to be significant in the univariate analysis were included in a full model with all potentially important social covariates and were adjusted for confounders. Finally, the odds ratio (OR) was calculated with 95% CI. A *P* value of <.05 was considered to yield statistical significance.

### Ethical Considerations

Ethical clearance was obtained from KCMC Research Ethical Committee. Informed consent was obtained from each mother prior to study participation. All mothers recruited were voluntarily participated in the study and signed an informed consent form. Participation in the study did not change the services rendered to PMTCT mothers in the clinic. Hospital file numbers were used instead of mothers' names to keep the confidentiality of any information provided.

## RESULTS

### Characteristics of the Study Participants

A total of 128 mothers living with HIV were recruited in this study. The age range of the mothers was 19 to 49 years with a mean ± standard deviation (SD) of 31 ± 6 (**Table 1**) and the mean age 6 SD of their infants during interview was 8.2 ± 2.5 months. All mothers had formal education, 87 (68%) of whom had received up to primary education. About three quarters (n=93, 72.7%) were Christians and the rest were Muslims. Of the study population, over half (n=72, 56.3%) were from urban areas, and the rest from the rural areas. A majority (n=107, 83.6%) lived in nuclear family – defined as a mother, father and their own children – and the rest lived in a joint family – defined as an extended family consist of more than 1 family or generation. Over half (n=66, 51.6%) were self-employed, about two-fifths (n=51, 39.8%) were housewives, and the remaining were employed. Almost three-fifths (n=74, 57.8%) of the participants reported their monthly income to be less or equal to 150,000 Tanzanian shillings (<US$100) and the remaining reported a monthly income of more than 150,000 Tanzanian shillings. About 46 (35.9%) of the respondents indicated that they were the sole income generators for their families, and 44 (34.4%) said they generated income with their partners. A majority (n=89, 69.5%) of the mothers interviewed were either married or cohabiting, almost a quarter (n=23, 18.0%) were single, and the remaining were either divorced or separated. More than three quarters (78.1%) were multiparous – having had multiple births – with a mean number of 3 infants.

**TABLE 1.** Characteristics of Study Participants (N=128)

Variable	Attribute	n (%)
**Mother's age (years)**	Mean (±SD, range)	31 (±6, 19–49)
	18–25	22 (17.2)
	26–35	79 (61.7)
	36–49	27 (21.1)
**Child's age (months)**	Mean (±SD, range)	8.2 (±2.5, 6–12)
	6–9	86 (67.2)
	10–12	42 (32.8)
**Mother's parity**	Mean (±SD, range)	3 (1, 1–7)
	Primipara	28 (21.9)
	Multipara	100 (78.1)
**Mother's education**	Up to primary	87 (68.0)
	Secondary	36 (28.1)
	Above secondary	5 (3.9)
**Mother's marital status**	Single	23 (18.0)
	Married/co-habiting	89 (69.5)
	Divorced/separated	16 (12.5)
**Mother's occupation**	Formal employment	11 (8.6)
	Self-employment	66 (51.6)
	Not employed/housewife	51 (39.8)
**Self-reported monthly income (T.Shs)**	Up to 150,000	74 (57.8)
	More than 150,000	54 (42.2)
**Type of family**	Nuclear	107 (83.6)
	Joint	21 (16.4)
**Main income provider**	Self	26 (20.3)
	Husband	46 (35.9)
	Husband + self	44 (34.4)
	Other family member	12 (9.4)
**Religion**	Christian	93 (72.7)
	Muslim	35 (27.3)
**Residence**	Rural	56 (43.7)
	Urban	72 (56.3)

### Other HIV-Related Concerns

Among the 100 multiparous mothers, 26.0% had another child infected with HIV. Of the 128 total respondents, the majority (89.1%) had had at least 4 antenatal care (ANC) visits. Almost three-quarters (n=94, 73.4%) of the mothers said they learned about their HIV status during the current child's pregnancy; only 16 (17.0%) of whom knew their status before they became pregnant, while others found out during previous pregnancies. All mothers delivered at a health facility. Of 128 infants, 113 (88.3%) were born with normal birth weight, while the remaining 15 (11.7%) were born low birth weight (**Table 1**).

### HIV-Status Disclosure

**FIGURE 1.**
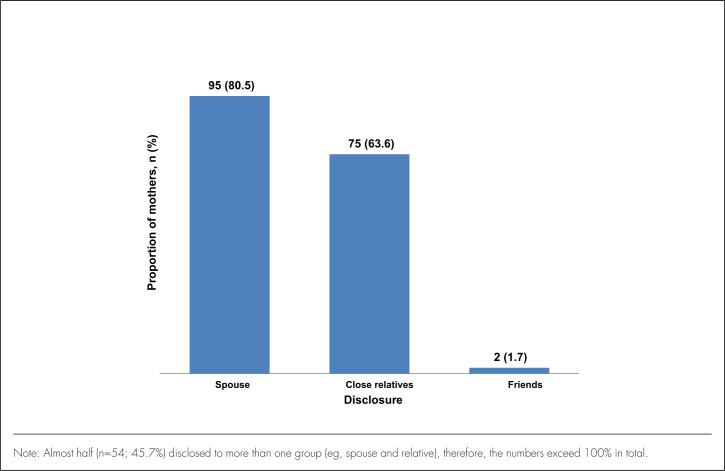
Disclosure of HIV Status to Any Person (N=118)

Note: Almost half (n=54; 45.7%) disclosed to more than one group (eg, spouse and relative), therefore, the numbers exceed 100% in total.

Of the 128 respondents, 118 (92.2%) had disclosed their HIV status: 95 (80.5%) to their spouses, 75 (63.6%) to their close relatives, and only 2 (1.7%) to their friends (**Figure 1**). Fifty-four (45.7%) of 118 mothers indicated that they had disclosed to more than 1 group of people. A majority (89.0%) of the mothers stated they disclosed before this birth and only 13 (11.0%) disclosed after birth of the last child.

### Feeding Counselling During Antenatal and Postnatal Care Visits

**FIGURE 2.**
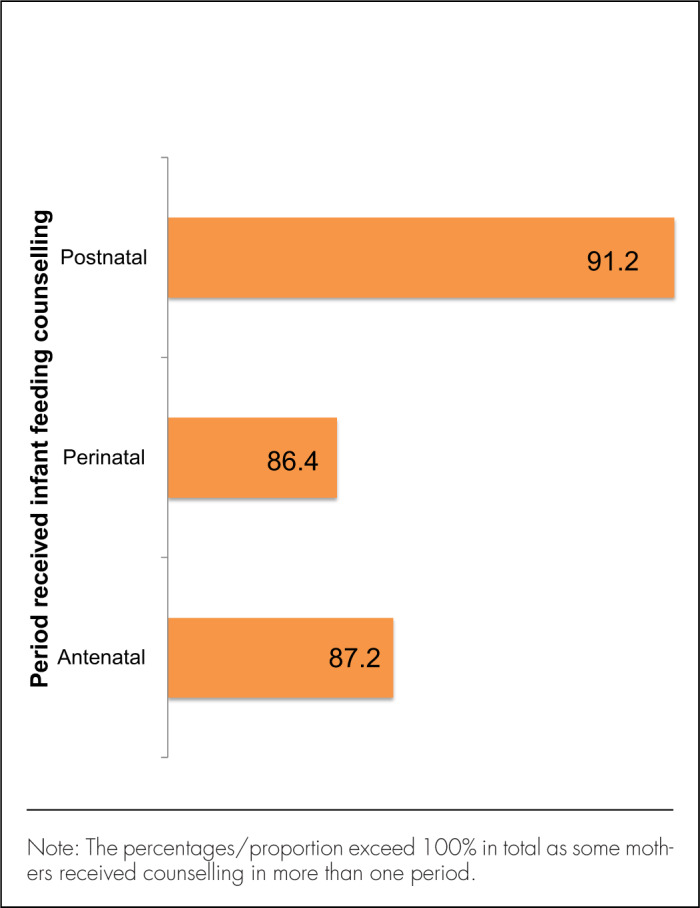
Infant-Feeding Counselling to Mother at Different Periods of Care (N=125)

Note: The percentages/proportion exceed 100% in total as some mothers received counselling in more than one period.

**FIGURE 3.**
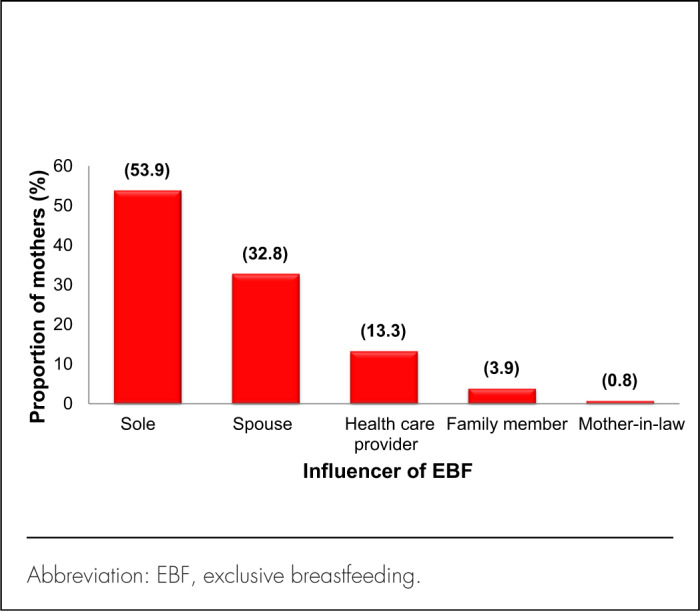
Person With Influence on Decision to Practice Exclusive Breastfeeding (N=128)

Abbreviation: EBF, exclusive breastfeeding.

Among 128 mothers, all but 3 had received feeding counselling at any stage during pregnancy and after delivery. About three-quarters (n=94, 75.2%) received counselling at all 3 stages – antenatal, perinatal, and postnatal periods, while 19 (14.8%) received counselling during 2 of the stages and the remaining 12 (9.4%) received counselling at only 1 stage (**Figure 2**). Over a quarter (27%) of those married or cohabiting reported that their partners attended the counselling sessions. More than 85% reported that they had discussed EBF with their partner and that the partner agreed to support EBF. More than half (n=69, 53.9%) reported that the decision to do EBF was solely theirs (**Figure 3**), while others were influenced by their spouse (n=42, 32.8%), health-care provider (n=17, 13.3%), family members (n=5, 3.9%), or mother-in-law (n=1, 0.8%). Of the 59 mothers influenced by other people to choose EBF, 5 were influenced by more than 1 person.

### Adherence to Exclusive Breastfeeding

Among the total 128 respondents, 71 (55.5%) reported they exclusively breastfed their children for the first 6 months of their child's life. No maternal characteristics showed a significant association with adherence status to EBF. Mothers who were the sole source of family income were more likely to adhere compared with those mothers who were dependent on spouses and relatives for their daily needs. This was the only factor showed statistical significance (*P*=.014) in univariate but not in bivariate analysis (**Table 2**).

**TABLE 2.** Selected Maternal Characteristics and Their Adherence Status With Exclusive Breastfeeding (N=128)

		Adherence Status			
Variable	Total	Adherent	Non-adherent	*P* Value	OR (95% CI)
		n (%)	n (%)		
Age (years)					
25 or younger	22	12 (54.5)	10 (45.5)		
Older than 25	106	59 (55.7)	47 (44.3)	.924	1.0 (0.4–2.4)
Education level					
Up to primary	87	47 (54.0)	40 (46.0)		
Post-primary	41	24 (58.5)	17 (41.5)	.632	0.8 (0.4–1.8)
Marital status					
Single/Divorced	39	25 (64.1)	14 (35.9)		
Married/Cohabiting	89	46 (51.7)	43 (48.3)	.193	1.7 (0.8–3.6)
Occupation					
Formally employed	11	7 (63.6)	4 (36.4)		
Not formally employed	117	64 (54.7)	53 (45.3)	.754	1.4 (0.4–5.2)
Place residence					
Rural	56	31 (55.4)	25 (44.6)		
Urban	72	40 (55.6)	32 (44.4)	.982	1.0 (0.5–2.0)
Parity					
Primipara	28	14 (50.0)	14 (50.0)		
Multipara	100	57 (57.0)	43 (43.0)	.510	0.8 (0.3–1.7)
Family income generation					
Self	26	20 (76.9)	6 (23.1)		
Others	102	51 (50.0)	51 (50.0)	.014*	3.3 (1.2–9.0)
Income per month (T-Shs)					
Up to 150,000	74	46 (62.2)	28 (37.8)		
More than 150,000	54	25 (46.3)	29 (53.7)	.074	1.9 (0.9–3.9)
Type of Family					
Nuclear	107	62 (57.9)	45 (42.1)		
Joint	21	9 (42.9)	12 (57.1)	.203	1.8 (0.7–4.7)
Number of antenatal visits					
Less than	4	13 7 (53.8)	6 (46.2)		
At least 4	115	64 (55.7)	51 (44.3)	.902	0.9 (0.3–2.9)
HIV status of siblings (n=100)					
Infected	26	11 (42.3)	15 (57.7)		
Not infected	74	46 (62.2)	28 (37.8)	.079	0.4 (0.2–1.1)
Counseling status					
Inadequate	19	13 (68.4)	6 (31.6)		
Adequate	109	58 (53.2)	51 (46.8)	.218	1.9 (0.7–5.4)
Disclosure status					
Not disclosed	10	6 (60.0)	4 (40.0)		
Disclosed	118	65 (56.1)	53 (44.9)	1.000	1.2 (0.3–4.6)
Influence to choice of EBF					
Own	69	41 (59.1)	28 (40.6)		
Other person's	59	30 (50.8)	29 (49.2)	.331	1.4 (0.7–2.9)

Abbreviations: CI, confidence interval; EBF, exclusive breastfeeding; OR, odds ratio.

Almost half (n=57, 44.5%) of infants were switched to other forms of feeding before the age of 6 months. The mean age ± SD was 3.32 months ± 1.24. Of the 57 infants, 52 (91.2%) stopped breastfeeding completely, while the remaining received MBF. Of the 52 infants who were stopped breastfeeding completely, 41 (78.8%) were given cow's milk and maize/millet porridge and 11 (21.2%) were given formula milk alone or with porridge.

### Barriers for Non-Adherence to Exclusive Breastfeeding

**FIGURE 4.**
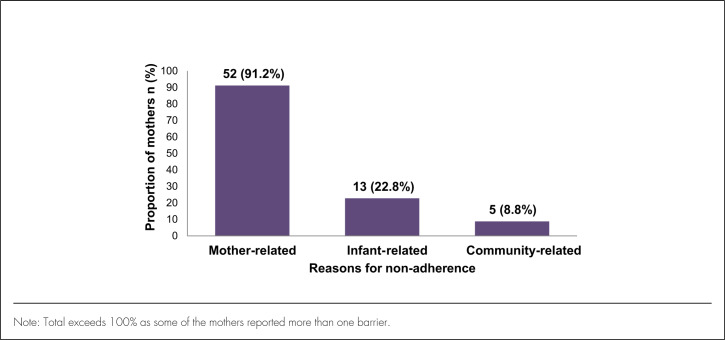
Barriers for Non-Adherence to Exclusive Breastfeeding (N=57)

Note: Total exceeds 100% as some of the mothers reported more than one barrier.

Barriers to adherence to EBF were divided into mother-, infant-, and community-related factors. Mother-related barriers for non-adherence of EBF were the most common (91.2%). Five of the most common responses by the mothers were: fear of HIV transmission to the infant through breastfeeding (67.3%), inadequate breast milk production (48.1%), mother had planned to breastfeed for 3 months only (23.1%), mother had to resume/go back to work (19.2%), and mother wished to stop after the first polymer-ase chain reaction (PCR) result was negative (15.4%) in order to avoid further exposure to breast milk. The most common infant-related responses given by study participants were increased demand of milk by the infant (46.1%) and 38.5% complete refusal of breast milk. Only 5 (8.8%) of the study participants gave community-related reasons, the most common of which was the tradition of early weaning (n=3, 60.0%) (**Figure 4**).

## DISCUSSION

In this study, 55.5% of mothers living with HIV adhered to EBF for the first 6 months. This timeframe was recommended by both WHO and the Tanzania Ministry of Health and Social Welfare (MOHSW) for feeding HIV-exposed infants.^7,14^ This finding was lower compared to those reported in Ethiopia (83.7%) and Nigeria (68.3%)^15,16^; and higher than those in India (44.0%), Guinea (15.5%), Uganda (24.0%), and Dar es Salaam, Tanzania (13.3%).^10,17–20^ The difference between this finding and that of the Dar es Salaam study can be explained by the involvement of several health facilities in the latter study. Information provided during feeding counselling may have differed at each health facility, depending on the counsellors' experiences, which may have contributed to the lower prevalence of EBF.^10^ The higher rate of EBF in our site may also be explained by the support provided during the frequent follow-up visits by ongoing counselling to mothers on infant-feeding practice done by counsellors at our centre. The lower proportion in India could be due to higher non-disclosure to partners and relatives and insufficient breast milk production, forcing mothers to wean early. In Uganda, the difference may be due to the tradition of prelacteal feeding – before breastfeeding is established – and the early introduction of staple foods, which was not the case in our study. The higher proportion in Ethiopia and Nigeria could be due to the study design comparing EBF with replacement feeding. The differences in adherence could also be explained by different social and cultural factors related to infant feeding.

In our study, the mean age for stopping EBF for mothers who did not adhere was 3.32 months. This was similar to that reported by Leshabari et al. and Young et al. in Tanzania.^9,10^ However, it was higher than the 1.8 months reported in a 2004 nationally general population representative sample^21^ and the 2.4 months in 2010 general population representative sample.^22^ This addresses that mothers still need more support and more adequate feeding counselling in order to follow the national recommendations of EBF for 6 months. The drop off by study participants after 3 months could be due to mothers resuming work after the national maternal leave of 84 days or that the mothers initially only planned to breastfeed for 3 months.

One-tenth of the mothers in our study who did not adhere to EBF practiced MBF and the remaining had stopped EBF completely. MBF is an unsafe mode of feeding and has shown to increase the risk of HIV infection fourfold compared to EBF.^8^ The proportion of MBF was lower than that reported in India (29.0%) and Zimbabwe (68.6%), and in a qualitative study conducted in the Kilimanjaro region with a small sample size involving only 13 mothers (100.0%).^8,9,17^ Our proportion was similar to that reported in Ethiopia and South Africa.^16,23^ The higher proportion in Zimbabwe could possibly be due to cultural norm to introduce liquids and solid foods very early life. In India, those mothers who practiced MBF may have done so because of insufficient breast milk, non-disclosure to their partners and relatives due to social repercussions, or their decision to change the mode of feeding. The lower proportion in this study may indicate that mothers might be provided with adequate knowledge on risks of MBF during feeding counselling in our centre.

While we acknowledge the low rate of MBF, some mothers stop breastfeeding completely before their infants are 6 months of age. Early cessation of breastfeeding can lead to serious gastroenteritis, which usually peaks at 3 to 4 months of life and is more severe if stopped during this period than when stopped later in life.^19^ In Tanzania, predictors of breastfeeding among mothers living with HIV in Dar es Salaam had shown that introduction of cow's milk at 4 months of age and becoming pregnant again contributed significantly to early cessation of breastfeeding.^20^ The tight clustering of stopping breastfeeding completely likely also reflects the effects of infant-feeding counselling. Material and social support can also contribute to reducing the number of mothers who stop breastfeeding earlier.

Surprisingly, maternal demographic characteristics were not statistically significant with adherence to EBF. Olandokun et al. in Nigeria had also seen no association between maternal characteristics and infant-feeding choices and practices.^18^ The association between mothers characteristics and infant-feeding patterns could have been neutralized by infant-feeding counselling at our centre or they may have been significant when mothers opted for replacement feeding, as reported in Ethiopia, because breastfeeding is norm in Africa.^16^ Mothers who were the sole income generators for the family adhered to EBF compared to mothers who were dependent on spouse and relatives. In Ethiopia, mothers who were daily labourers and generated income on their own were more likely to choose and adhere to feeding option in a recommended way.^16^ This higher level of EBF by sole earners may be due to the power these mothers have on make decisions on whether to adhere to EBF or not. In Nigeria, mothers who were more than 31 years of age adhered to the feeding option chosen and was proven significant.^15^ However, they only compared 2 modes infant feeding, and EBF was not an option.

Maternal characteristics were not significant but those who had post-primary education; were single or divorced, formally employed, multiparous, or living in a nuclear family; and whose monthly income was up to 150,000 Tanzanian shillings, were more adherent to EBF. This could be because mothers with a post-primary education or higher may have more easily understood the concept of EBF. The single or divorced group had good adherence, which may be explained by the fact that they make decisions on their own and not dependent on spouses. Multiparous mothers were more adherent; this could be due to their maturity and past experience. Those living in nuclear family were more adherent than those living in a joint family. This difference may be due to non-nuclear family pressures to add other foods in early life and to families deeply involved in culture and tradition.

Disclosure of HIV status had also shown no significance with adherence to EBF. While those who disclosed in Ethiopia and Nigeria were more likely to practice the recommended feeding option,^15,16^ this may be because in both studies some mothers had opted for replacement feeding. In Burkina Faso, partners of mothers who opted for replacement feeding and had disclosed were seen to support the mothers by making false statements, like having lesions on the breast to the community, if questioned why they were not breastfeeding.^24^ Disclosure status may, however, have no association with breastfeeding because breastfeeding is a norm in Africa.

Although the study did not set out to test the effectiveness of infant-feeding counselling status, counselling on feeding and adherence status had no significance to EBF; however, the proportion of mothers who adhered to EBF in our study was good, with low proportion of MBF. In Nigeria, mothers who had more than 1 counselling session during ANC adhered more to the feeding option, however, that study only compared 2 modes of feeding.^15^ This indicates that the more ANC visits the mother makes the more likely she is to adhere to the mode of feeding chosen. This is because they get enough time to discuss their concerns with the counsellors. The impact of EBF counselling programs for African mothers living with HIV should consider individual maternal, social, and health contexts. Therefore, counsellors at ANC clinics play a major role in infant-feeding counselling, particularly EBF. The Tanzania MOHSW and implementing partners have done much to strengthen the infant-feeding counselling aspect of PMTCT services.^6^ However, a large gap still remains in achieving adequate number and trained counsellors especially at ANC facilities.^25^ This gap is a concern for Tanzania as well as sub-Saharan Africa.

The 5 most common mother-related reasons given by the study participants were fear of transmitting HIV through breastfeeding, poor or inadequate milk production, mother had planned during ANC visit to feed for 3 months only, mother had to resume/go back to work after maternal leave, and mother chose to stop breastfeeding once she found that the infant's first PCR was negative. In Ethiopia, insufficient breast milk was found to be significant in practising feeding in a recommended way.^15^ Similar reasons were reported in Zimbabwe, where the primary barrier to mothers living with HIV continuing breastfeeding for the entire 6-month period was to protect their child from HIV infection. They worried less about infant health and survival associated with early cessation of EBF. Other reasons for stopping EBF were negative PCR results at 6 months of age and poor nutrition of the mother, which was indirectly related to inadequate milk production.^26^ However, there was no significance to HIV-free survival of infants of mothers who stopped EBF completely at 4 months compared to those who continued EBF and then complementary feeding.^27^ WHO recommends EBF to all mothers, regardless of the HIV status, and with continued breastfeeding for 2 years.^7^ These recommendations were also emphasized in the 2016 WHO breastfeeding guidelines, especially where counselling on adherence and provision of ARVs is provided.^12^ When counselling mothers living with HIV, it is important to identify barriers that could mitigate adherence to EBF.

Other common reasons reported by different countries in Africa for not adhering to EBF are: mother's level of education, mother always involved in household activities, stigma of HIV, local culture and/or traditions, and family pressure to add other foods, especially traditional gruels.^9,15,24,26^ In the current study, education was the least among the reasons followed by culture and/or tradition. This may be because a higher percentage of mothers had at least a primary education, which is completed at ages 13 to 15 years, and the mothers in this study were ages 19 years or older. Community-related barriers were mentioned by few mothers, this may be because most of the mothers in this study were living in a nuclear family, which usually means decisions are made by a woman and her husband, without pressure from mothers-in-law and other relatives.

### Limitations

There were few important limitations to this study. This was a hospital-based study in a tertiary-care hospital where counselling is expected to be of good quality, which might limit the generalization. Also, recall bias may be a possibility because infant-feeding information depended on mother self-reporting. However, we suspect majority of mothers could recall duration of exclusive breastfeeding and barriers associated accurately since the study enrolment was done while they were still practicing breastfeeding. The study did not perform assessment of the AFASS criteria to determine the appropriateness of replacement feeding in the infants who were never breastfed.

## CONCLUSION

Many of the mothers living with HIV who participated in our study did not adhere to recommendations to exclusively breastfeed their uninfected yet HIV-exposed infants for 6 months after birth. About 45.0% of the participants indicated that they did not adhere to recommended duration of EBF. Of this group, one-tenth practised MBF and the rest stopped breastfeeding completely. Of the reasons for non-adherence to EBF extrapolated from study participants, the most common reasons were mother related: fear of postnatal transmission of HIV through breastfeeding and poor milk production were the most common responses given. To support the 2016 breastfeeding guidelines, more emphasis should be put on mothers living with HIV to exclusively breastfeed their infants for the first 6 months of life and to continue breastfeeding while introducing complementary feeding.

As a result of our research, we suggest that more effective and intensive feeding counselling and additional psychological counselling and education on the risks of early cessation of EBF should be provided to mothers and their partners throughout pregnancy and after delivery. Further longitudinal research is needed to see the outcomes of HIV naive or positive mothers who do or do not adhere to EBF.
